# Perception among NHIS–HMO Enrolees of the Attitudes of Medical Personnel during Outpatient Care in Lagos Hospitals

**DOI:** 10.3390/ijerph20021218

**Published:** 2023-01-10

**Authors:** Abigail Affiong Mkperedem, Peter Ogunlade, Chisaa Igbolekwu, Ogadimma Arisukwu, Stephen Olugbenga Owa, Abiodun Olawale Afolabi, Stephen Otu Etta-Oyong

**Affiliations:** 1 Department of Sociology, Landmark University, Omu-Aran 251103, Nigeria; 2 Department of Food Science & Microbiology, Landmark University, Omu-Aran 251103, Nigeria; 3 Department of Agricultural Economics, Landmark University, Omu-Aran 251103, Nigeria; 4 Department of Extension and Rural Development, Landmark University, Omu-Aran 251103, Nigeria

**Keywords:** perception, national health insurance scheme, health maintenance organisation, medical personnel, quality, attitude

## Abstract

The quality of healthcare services depends on the interactions between administrators, customers, and healthcare providers. This study seeks to examine how National Health Insurance Scheme (NHIS)–Health Maintenance Organization (HMO) enrolees perceive the attitudes of medical personnel while receiving outpatient care in Lagos hospitals. Mixed methods were used, i.e., distributing questionnaires and in-depth interviews with participants. Quantitative data were analysed using Statistical Product and Service Solutions (SPSS) version 20, and approaches that involved frequency statistics, factor analysis, contingency chi-square and correlation analysis were applied. The results indicate that the variables “humane” and “empathetic” have a direct impact on enrolees’ perception and an indirect impact on motivation to adhere to medical advice among enrolees. This research has practical implications, especially in light of new initiatives of NHIS collaboration with HMO to deliver quality healthcare services to enrolees.

## 1. Introduction

Behaviour and attitudes related to healthcare strongly influence the quality of healthcare service delivery. The actions performed differ depending on the conditions under which care is provided, as well as on the relationships developed among the actors [1]. Although these relationships may vary from person to person in different care situations, they present something universal, allowing the patients who are a part of them to find meaning in terms of their present situation. Healthcare actions, although experienced personally, involve interpersonal relationships and are a major part of social life. According to the 1949 Geneva Convention and the 1977 Additional Protocols, ‘medical personnel’ are persons assigned exclusively to medical duties, whether such assignments are permanent or temporary [2].

Assessing the quality of healthcare personnel attitudes requires the establishment of a face-to-face relationship, defined in [3] as a social relationship built upon the expertise of the medical personnel involved in the promotion of health, leading to either positive or negative experiences on the part of the patient. Such relationships are between subjects who are mutually aware (in time and space). The authors of [4] considered that assessments of the quality of service of health-providing institutions could be divided into two categories.

The first component involves technical or clinical aspects, which comprise all technical diagnoses and procedures. The second is the functional aspect, which is defined by how the healthcare services are delivered to the consumer (for example, the mannerisms exuded by medical professionals during patient care, the structural and physical nature of the hospital, and the quality of meals served). According to the authors of [5], factors such as skills, knowledge, temperament, and so on heavily influence how healthcare personnel dispense care to consumers. In order to accommodate patients’ needs and preferences [6], healthcare practitioners need to be endowed with interpersonal qualities such as tolerance, technical know-how, sensitivity and confidentiality, empathy, and reliability [5].

The National Health Insurance Scheme (NHIS) and its collaboration with the private Health Maintenance Organization (HMO) is a Nigerian government initiative intended to ensure that all citizens have access to healthcare at a reasonable cost to achieve universal health coverage (UHC) nationwide. However, poor referral systems, excessive distance to facilities, episodic coverage, delays in processing authorisation codes, poor attitude/unprofessional conduct by healthcare personnel, and unequal access to services between public NHIS and private HMO enrolees have been noted as challenges facing the NHIS scheme in Nigeria’s enrolees [6,7,8,9].

Several studies have been conducted to determine which areas of healthcare services are most influential on the experiences of patients. For example, the authors of [10,11,12] noted that perceptions among patients may be based on their interactions with healthcare providers. This paper, therefore, focuses on how NHIS–HMO enrolees perceive the attitudes of medical personnel while receiving outpatient care in Lagos hospitals.

### 1.1. Theoretical Framework

#### Assessment of the Quality of Medical Care through the Lens of Social Phenomenology

The nature of the present research justified the adoption of the social phenomenology of Alfred Schütz (1899–1959) [13] to understand NHIS–HMO enrolees’ perceptions of medical personnel attitudes during outpatient care. Schütz posited that humans rely on communication and their intellectual know-how to actively participate in social acts. Social phenomenology essentially seeks to provide an understanding of how mutual relationships occur through human interactions, environmental influencing cues, and the construction of social reality.

The idea focuses on how individual actors interpret social actions to understand and interpret personal experiences [13,14]. From the perspective of Alfred Schütz, assessments of the quality of healthcare can be based on the multiple social actions that occur within such settings. An established interpersonal relationship supports the recognition of the knowledge and experience of the care provider within the hospital setting. It may be assumed that the healthcare facility is a place involving interactions between subjects (medical personnel and healthcare consumers). Therefore, such a setting must be recognised as a dynamic environment in which social interactions occur. 

One important implication of the social phenomenology in this study is that enrolees make sense of or characterize the attitudes of healthcare personnel, i.e., as being good or bad, based on the actions, situations, and realities that take place within the healthcare facility. This implies that there is a need for awareness and acceptance of the social behaviours of the relevant subjects in a hospital setting [14].

Social phenomenology assumes that the perception of the quality of medical care among enrolees will be guided by the social relations established in such settings [15]. This framework underlines the importance of interactive social relationships between those involved in healthcare actions as this has a major impact on perceptions among healthcare recipients. This theory was adopted to guide the present investigation in how the attitudes of medical personnel play an important role in the formation of healthcare recipients’ perceptions.

## 2. Materials and Methods

The study employed triangulation; hence, quantitative data were elicited using questionnaires and the qualitative data were captured using in-depth interviews (IDIs) [16].

### 2.1. Sample Size and Sampling Procedure

#### 2.1.1. Quantitative Sample Size Determination

Factor analysis of the subject-to-variable ratio with a minimum of ten (10) subjects per variable in the study instrument was utilized to choose a sample size of 240 enrolee respondents (20 subjects for each of the 12 variables in the study instrument). The minimum sample size of approximately 266 (240/0.9) patients was reached after adjusting for 10% non-response to the questionnaire [16,17,18].

#### 2.1.2. Study Population

The study population was composed of enrolees visiting selected hospitals in Lagos. The accredited public and private HCFs included: (1) St. Mary Specialist Hospital, (2) Awoyaya Hospital, (3) Blue Cross Hospital, (4) Unity Hospital, (5) The Eko Hospital, (6) General Hospital Akodo, (7) Budo Specialist Hospital, (8) Etta Atlantic Memorial Hospital, and (9) St. Nicholas Hospital [16].

#### 2.1.3. Justification for HCPs/HCFs Selection

The sample hospitals were chosen through the ballot system from a list of registered healthcare providers (HCPs) across the senatorial districts in Lagos state available on the NHIS website. To depict the two dominant healthcare facility systems in Nigeria, the selection included both public and private hospitals [16].

#### 2.1.4. Quantitative Sampling Technique

**Figure 1:**
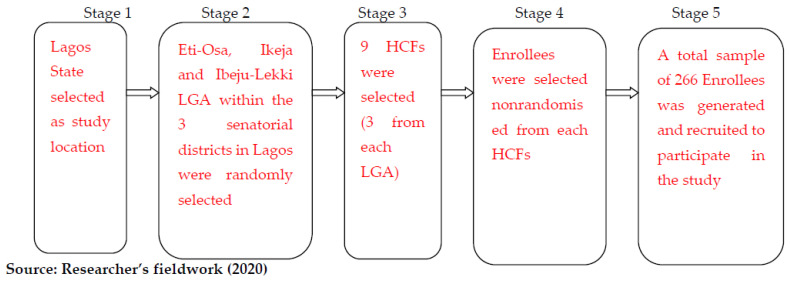
Multistage sampling approach for study area and study participants.

As shown in Figure 1 [16], a multistage sampling technique was used to select the study participants. Simple random sampling was employed at each stage to reduce selection bias.

In stage 2, the twenty (20) local governments were clustered into the three senatorial districts, selecting only one local government from each district through balloting.

Stage 3 involved obtaining a list of all registered HCFs within the local governments and stratifying them into private and government-administered [16]. It also involved the selection of 9 HCFs from the larger pool through a ballot system. At this point, every HCF had the same probability of being chosen to be sampled in the study.

#### 2.1.5. Validity and Reliability of the Research Instrument

The construct and content validities are applicable in this study. To guarantee the validity of the study, the research instrument was carefully structured in line with the study objective. The content of the instrument was compared with available works of the literature on the topic. Items in the questionnaire, the appropriateness of language, and instructions to the respondents were reviewed and corrected by the study supervisors [16]. The internal consistency of the instrument was determined by a pre-test on 25 enrolees and 6 HCFs in the study location.

#### 2.1.6. Method of Data Collection

The data-collection methods employed for this study were a questionnaire for quantitative data and IDI for qualitative data. Both qualitative and quantitative methods were used to enhance the validity of results through triangulation. To this end, both interview and questionnaire administration were conducted physically at the same time in selected healthcare facilities in Lagos. The fieldwork for the study was conducted between August and December 2020. Data were collected in English and Yoruba (the most commonly spoken local languages in Lagos state). The questionnaire items were interpreted for responders who were not able to communicate in English by the Research Assistants [16].

### 2.2. Research Instruments

#### Quantitative Data Collection Instrument

To determine how enrolees perceived the quality of medical personnel attitudes during outpatient care in the selected hospitals, a 24-item structured questionnaire divided into three sections—demographic data, attitude of medical personnel during outpatient care, and satisfaction perception—were administered to NHIS–HMO enrolees who visited the selected HCFs during the time of the study.

### 2.3. Questionnaires and Scales

#### 2.3.1. Attitude Questionnaire

The constructed variables to determine quality measures on medical personnel attitude were performed by adjustment [17,18]. The quality indicator variables were presented according to a five-point Likert scale position ranking [19]. All questions had response options rating variables on a five-point Likert scale (1 = strongly disagree, 2 = disagree, 3 = not sure, 4 = agree, 5 = strongly agree). During hypothesis testing, these ordered categories were transformed, and the responses were converted into five (5) categories termed: five (very good), four (good), three (undecided), two (bad), and one (very bad). The transformation was performed for ease of comparison and interpretation [16,19].

#### 2.3.2. Self-Efficacy for Attitude Perception Scale

Enrolees were asked to indicate, under several different circumstances, their level of satisfaction with the attitudes of medical personnel during outpatient care on a three-point response scale (1 = bad attitude perception, 2 = average attitude perception, 3 = good attitude perception). Good perception was considered the optimal level while bad and average perceptions were considered suboptimal levels of attitude satisfaction. The contingency Chi-square and Spearman’s correlation coefficient (*r*) were used to analyse the elicited data.

### 2.4. Qualitative Data Collection Instrument

#### In-Depth Interview (IDI)

To gain a deeper understanding of the enrolees’ perception of the quality of service and encourage them to tell ‘the story’ in their own words, in-depth interviews were conducted with 10 selected enrolees. All the participants were selected based on their availability at the time of tracing and willingness/ability to provide written informed consent/assent. To facilitate analysis, interviews were recorded digitally with the participants’ permission and notes were taken for participants who were reluctant about voice recording.

### 2.5. Method of Data Analysis

#### 2.5.1. Quantitative Data Analysis

The quantitative data collected in this research were analysed using the nominal descriptive statistics of frequencies and simple percentages with the help of the Statistical Product and Service Solutions (SPSS) version 20. The hypothesis was tested with the contingency Chi-square and Spearman’s correlation analysis. These tools were chosen due to the ordinal nature of the data. To ensure adequateness, completeness, legibility, and consistency, the questionnaire was edited before the entry of the data into the system. Enrolees’ socioeconomic characteristics were analysed using descriptive statistics and results presented in frequency distribution and percentages. Enrolees’ perceptions of healthcare service quality were analysed using Spearman’s correlation coefficient (*r*). This is because the variables were taken from ordinal scales. Additionally, correlation analysis was used to reveal meaningful relationships between the two variables of the study (enrolees’ perception and healthcare services).

#### 2.5.2. Qualitative Data Analysis

Using Donabedian’s [20] process element, inductive content analysis known as hermeneutics was used in the analysis of the qualitative data. Content analysis is a common term for several different strategies used to analyse text [16].

### 2.6. Ethical Approval and Informed Consent

This study was approved by the Lagos State Government Health Service Commission under approval code number LSHSC/88/S.3/II/257. The study fieldwork was supervised by all healthcare facilities/healthcare providers. All participants signed the informed consent form. The analyses reported here were performed in accordance with relevant guidelines and regulations [16].

## 3. Results

The majority (67.5%) of the respondents were female, which corresponds with the country’s last census report. A larger proportion (53.2%) of the respondents was married than not. The majority (36.0%) of the respondents fell within the age bracket of 31 and 40, which represents the active working population with a mean interval of 3.0278. Although more expensive, a larger proportion of the respondents (81.3%) subscribed to the private HMO and 82.5% accessed care in private HCFs [16], as observed in Table 1.

In Table 2 below, the majority (69.8%) combined weight of Strongly Agree (SA) and Agree (A) indicates that the overall attitude of medical personnel during outpatient care was humane and respectful. While a 69.8% combined weight of Strongly Agree (SA) and Agree (A) shows the factoring of respondents’ medical history into treatment, a 35.3% combined weight of Disagree (D) and Strongly Disagree (SD) shows that respondents were not motivated by the attitudes of medical personnel.

Responses from the care element during the IDI, however, negated the quantitative result. When probed to elaborate on what was considered inclusive of humane treatment, an interviewee motioned:

“The level of empathy from the healthcare workers beginning from the gate is so appalling”.

(IDI 4. Male, 60)

Another interviewee lamented:

“I am still in pain, but what can I do? If you complain, you get delayed, or you are labelled a troublemaker”.

(IDI 1. Female, 25)

Another interviewee noted:

“The medical facility needs to employ more personnel or refer to other facilities to ensure personnel do not feel burdened as this is obvious in the way they tend to relate with us, the patients”.

(IDI 3. Female, 28)

Taking into consideration all other factors relating to the attitude of medical personnel, respondents’ perceptive rating of medical personnel reveals that more than half (53.6%) perceived the attitude of medical personnel to be good; however, a significant 30.5% of the enrolee respondents also perceived the attitude of medical personnel during outpatient care to be bad, as shown in Table 3.

### Test of Hypothesis

**H_0_.**  *There is no significant relationship between the quality of medical personnel attitudes and enrolees’ perception.*

**H_1_.**  *There is a significant relationship between the quality of medical personnel attitudes and enrolees’ perception.*

Decision criterion: Reject H_0_ if the calculated (observed value) of chi-square (χ^2^c) is found to be greater than the critical (table) value of chi-square χ^2^t (0.01); if not, do not reject. Data from statement three (3) in Table 2 and Table 3 were cross-tabulated and used in testing this hypothesis. The result is shown in Table 4.

Table 4 shows the relationship between the attitudes of medical personnel during outpatient care and enrolees’ perception. A total of ninety-one (91) respondents’ who had a good perception and also saw the attitude of medical personnel as very good and good is higher than those (32) who were very high in perception but saw the quality of medical personnel as bad and very bad. Moreover, the 91 respondents with a good or higher perception is higher than those (37) who were very low in perception and saw the quality of medical personnel as very bad and bad. Empirically, the result from group comparisons reveals an existing relationship between medical personnel attitudes and enrolees.

Inferential statistics also support this observation because the calculated χ^2^ (16) = 82.265 is higher than the chi-square table (*p* > 0.01). Therefore, the null hypothesis, which states ‘there is no significant relationship between quality of medical personnel’ attitude and enrolees’ perception’, is rejected, and the alternate hypothesis corroborating that ‘there is a significant relationship between quality of medical personnel attitude and enrolees’ perception’ is accepted. Moreover, the correlation was found to be significant at the 0.01 level as Spearman’s correlation *(r*) = 0.219 shows a positive relationship between the quality of medical personnel attitudes and enrolees’ perception.

## 4. Discussion

During the IDI, the quality of medical personnel’s attitude was noted to be time and responsibility dependent. Likewise, the researchers observed that irregular personality traits and a shortage of medical personnel in some of the HCFs could have affected the attitudes of medical personnel. While this was a commendable effort by government, the critical shortage of skilled health workforce evident in sub-Saharan Africa [18] has overwhelmed the human resources for improved health indices. This finding is in consonance with the results of [5,6].

Observational analysis of enrolees’ perception of medical personnel attitudes during IDI showed that care must go beyond the procedure and take into account the human essence by possessing characteristics such as kindness, promptness, respect, and empathy. It will be good, therefore, to take into account the attitudes of medical personnel to prevent a decline in health indices while sustaining essential services in the healthcare system. This finding is consistent with the social phenomenology ideology [13,14] and Donabedian’s [20] process element.

The aim of this paper was to describe how enrolees perceived the quality of medical personnel attitudes during outpatient care in Lagos hospitals. The hypothesis formulated for this objective was that ‘there is no significant relationship between quality of medical personnel attitudes and enrolees’ perception’. Questions asked in the questionnaire generated the data presented in Table 1, Table 2 and Table 3. While the majority (69.8%) of respondents indicated that the overall attitude of medical personnel was humane and respectful during outpatient care, 35.3% reacted negatively to the question regarding motivation by medical personnel attitudes to follow prescribed treatment. Similar results have also been reported in a previous study [4].

This study’s findings showed that the overall attitude of medical personnel during outpatient care was humane and respectful. This follows the recommendations of [1,2]. Data from Table 2 and Table 3 were cross-tabulated to test the hypothesis. The contingency chi-square test (*p* < 0.01, χ^2^ (16) = 82.265) and Spearman rank correlation coefficient analysis (0.219) in Table 4 indicated a significant relationship between medical personnel attitudes and enrolees’ perception. Therefore, the null hypothesis was rejected, and it was concluded that a significant association between the quality of medical personnel attitudes and enrolees’ perception existed. This relationship assumes the ideas of [5,12,13,20].

Some of the respondents in the IDI expressed varying perceptions regarding the quality of medical personnel attitudes, which was dependent on time and crowd proportion. Similar results have also been reported in previous studies [6,7,8,9]. From IDI responses, it is evident that respondents perceived the varying medical personnel attitudes to be a result of varying factors, such as experience, individual abilities, and personality differences [6,11].

The authors of [6,8] indicated that a major element influencing patients’ perceptions of medical personnel performance is tangibility. Enrolees’ opinions of medical personnel are strongly influenced by the outward behaviour of personnel and face-to-face interactions [2,20]. The authors of [6,7,8,9] suggested that variables related to medical personnel attitudes towards NHIS enrolees are related to socioeconomic and healthcare plans. The IDI finding was also consistent with enrolees’ low-level satisfaction with medical personnel attitudes in Ghana [21] and Kallu, Ethiopia [22].

### 4.1. Strength and Limitations of the Study

Attention has been paid to other stakeholders of the NHIS–HMO program, the extent of enrolment, and the quality of services rendered under the scheme while evaluating healthcare quality. The findings of this study contribute to existing knowledge by exploring the perception of the NHIS–HMO enrolees as influenced by the quality of medical personnel attitudes during outpatient care in one of the world’s fastest-growing cities—Lagos, Nigeria. Additionally, by examining the public–private partnership (NHIS–HMO), multiple HCFs and the two types of medical care, this study has demonstrated significant enrolee perceptions of the quality of medical personnel attitudes during outpatient care across the Lagos healthcare system.

- The study was limited to enrolees visiting the selected hospitals for outpatient care not inclusive of enrolees who were present at the selected hospitals for inpatient care during the study period.
- The study was also limited to enrolees visiting selected HCFs in Lagos State; therefore, the result is not relevant to the other thirty-five (35) states in Nigeria.
- The qualitative study was limited to IDI.
- The qualitative method of analysis was limited to inductive content analysis while the quantitative was limited to Chi-square and Spearman’s correlation analysis.

### 4.2. Prospect for Future Research

- The same study should be replicated in the other seventeen (17) local government areas of the state, and the results should be compared.
- Research should also be conducted to find out the determining factors responsible for enrolees’ choice of hospital utilization and its consequences on the perceived quality of medical personnel attitudes.
- Considering the methods of analysis in this study, studies should be conducted with other methods of analysis for the purpose of replicability.
- Furthermore, a comparative study should be carried out on the same study.

## 5. Conclusions

The research findings reveal that the quality of medical personnel attitudes during outpatient care has a significant relationship with enrolees’ perception. This implies that perception may be altered depending on the attitude displayed upon the next visit to the same or a different hospital. Following the sharp contrast between the questionnaire reports and the IDI, the research further concludes that enrolees’ perception is sharpened by their experience or the experience of others witnessed or heard.

Moreover, the study findings indicate that the intangible humane and respectful attitudes of medical personnel can serve as a motivating factor for patient to adhere to medical instructions. This also concludes that during access and utilization of healthcare, enrolees take into cognizance both tangible and intangible aspects of care. Conclusively, while facilitating the achievement of the World Health Organization (WHO) “Health for All” targeted by the year 2030, hospital management should only accept enrolees proportionate with the capacity of medical personnel to ensure quality service that guarantees positive professional medical attitudes at all times. 

## Figures and Tables

**Table 1:** Sociodemographic characteristics of respondents.

Variable	Label	Frequency (N = 252)	Percentage (%)
Marital Status	Single	70	27.8
	Married	134	53.2
	Other	48	19.0
Sex	Male	82	32.5
	Female	170	67.5
Age	18–20	22	8.7
	21–30	66	26.2
	31–40	93	36.9
	41–50	25	9.9
	51–65	46	18.3
Educational Qualification	No Formal Education	22	8.7
	First Leaving School Certificate	15	6.0
	Secondary School	34	13.5
	OND/NCE	32	12.7
	HND/B.Sc	101	40.1
	M.Sc/MBA/M.Ed	38	15.1
	Ph.D.	10	4.0
Public–Private Partnership Enrolment	Public NHIS	47	18.7
	Private HMO	205	81.3
Healthcare Facility/Healthcare Provider (HCFs/HCPs) Utilization	Public/Government HCFs/HCPs	44	17.5
	Private HCFs/HCPs	208	82.5

**Table 2:** Distribution of respondents’ perception of the attitude of medical personnel.

Variable	Responses	Frequency	Percentage (%)
The medical personnel’s attitude during my outpatient care was humane and respectful	Strongly Agreed	79	31.3
	Agreed	97	38.5
	Undecided	1	0.5
	Disagree	49	19.4
	Strongly Disagree	26	10.3
	Total (%)	252	100
My medical history (previous illnesses and family history) was factored in during treatment	Strongly Agreed	62	24.6
	Agreed	114	45.2
	Undecided	18	7.1
	Disagree	47	18.7
	Strongly Disagree	11	4.4
	Total (%)	252	100
The medical personnel’s attitude motivated me to follow the treatment prescribed.	Strongly Agreed	47	18.7
	Agreed	87	34.5
	Undecided	29	11.5
	Disagree	33	13.1
	Strongly Disagree	56	22.2
	Total (%)	252	100

**Table 3:** Distribution of respondents’ perceptions of medical personnel attitudes.

Variable	Perception	Frequency	Percentage (%)
Respondents’ ratings of medical personnel attitude	Good	135	53.6
	Average	40	15.9
	Bad	77	30.5
	Total	252	100

**Table 4:** Relationship between attitudes of medical personnel during outpatient care and enrolees’ perception.

Response	Good	Average	Bad	Total	χ^2^
	Perception (%)	Perception (%)	Perception (%)		
Attitude					
Very Good	24 (29.8)	7 (14.9)	16 (34)	47 (100.0)	χ^2^ = 82.265
Good	67 (77)	8 (9.2)	12 (13.8)	87 (100.0)	*r* = 0.219
Neutral	12 (41.3)	5 (19.4)	12 (41.4)	29 (100.0)	*p* = 0.000
Bad	12 (36.4)	7 (17.2)	14 (42.4)	33 (100.0)	df = 16
Very bad	20 (35.7)	13 (21.2)	23 (41.1)	56 (100.0)	
Total	135 (53.6)	40 (15.9)	77 (30.6)	252 (100.0)	

## Data Availability

Not applicable.
